# Coupled Carbon, Sulfur, and Nitrogen Cycles Mediated by Microorganisms in the Water Column of a Shallow-Water Hydrothermal Ecosystem

**DOI:** 10.3389/fmicb.2018.02718

**Published:** 2018-11-13

**Authors:** Yufang Li, Kai Tang, Lianbao Zhang, Zihao Zhao, Xiabing Xie, Chen-Tung Arthur Chen, Deli Wang, Nianzhi Jiao, Yao Zhang

**Affiliations:** ^1^State Key Laboratory of Marine Environmental Science, Xiamen University, Xiamen, China; ^2^College of Ocean and Earth Sciences, Xiamen University, Xiamen, China; ^3^Department of Limnology and Bio-Oceanography, University of Vienna, Vienna, Austria; ^4^Department of Oceanography, National Sun Yat-sen University, Kaohsiung, Taiwan

**Keywords:** shallow-water hydrothermal ecosystem, Kueishantao Islet, metatranscriptomics, 16S rRNA library, microbial community, metabolic pathway, biogeochemical cycle, coupling

## Abstract

Shallow-water hydrothermal vent ecosystems are distinctly different from deep-sea vents, as other than geothermal, sunlight is one of their primary sources of energy, so their resulting microbial communities differ to some extent. Yet compared with deep-sea systems, less is known about the active microbial community in shallow-water ecosystems. Thus, we studied the community compositions, their metabolic pathways, and possible coupling of microbially driven biogeochemical cycles in a shallow-water hydrothermal vent system off Kueishantao Islet, Taiwan, using high-throughput 16S rRNA sequences and metatranscriptome analyses. Gammaproteobacteria and Epsilonbacteraeota were the major active bacterial groups in the 16S rRNA libraries and the metatranscriptomes, and involved in the carbon, sulfur, and nitrogen metabolic pathways. As core players, *Thiomicrospira, Thiomicrorhabdus, Thiothrix, Sulfurovum*, and *Arcobacter* derived energy from the oxidation of reduced sulfur compounds and fixed dissolved inorganic carbon (DIC) by the Calvin-Benson-Bassham (CBB) or reverse tricarboxylic acid cycles. Sox-dependent and reverse sulfate reduction were the main pathways of energy generation, and probably coupled to denitrification by providing electrons to nitrate and nitrite. Sulfur-reducing Nautiliaceae members, accounting for a small proportion in the community, obtained energy by the oxidation of hydrogen, which also supplies metabolic energy for some sulfur-oxidizing bacteria. In addition, ammonia and nitrite oxidation is another type of energy generation in this hydrothermal system, with marker gene sequences belonging to Thaumarchaeota/Crenarchaeota and *Nitrospina*, respectively, and ammonia and nitrite oxidation was likely coupled to denitrification by providing substrate for nitrate and nitrite reduction to nitric oxide. Moreover, unlike the deep-sea systems, cyanobacteria may also actively participate in major metabolic pathways. This study helps us to better understand biogeochemical processes mediated by microorganisms and possible coupling of the carbon, sulfur, and nitrogen cycles in these unique ecosystems.

## Introduction

The discovery of marine hydrothermal vents greatly enhanced our understanding of microbial habitats and survival strategies as well as the origins of life. Microbial communities in deep-sea hydrothermal systems have been intensively studied (Brazelton and Baross, 2010; Xie et al., 2011; Grosche et al., 2015; Anantharaman et al., 2016) since the discovery of these vents in 1977. Most microbes in deep-sea hydrothermal vent ecosystems carry out chemosynthesis, which fixes carbon dioxide (CO_2_) into organic compounds using the energy released by chemical reactions; it does not require sunlight. However, in shallow-water hydrothermal vent ecosystems, generally at water depths less than 200 m, chemolithoautotrophy and photoautotrophy occur simultaneously (Maugeri et al., 2009; Zhang et al., 2012; Gomez-Saez et al., 2017; Tang et al., 2018). Previous surveys of bacterial 16S rRNA genes using tag pyrosequencing and clone libraries revealed a high abundance of chemoautotrophs within the classes Gammaproteobacteria and Epsilonproteobacteria (reclassified to a new phylum Epsilonbacteraeota; Waite et al., 2017) in shallow-water hydrothermal systems (Tang et al., 2013; Gomez-Saez et al., 2017). In addition, Cyanobacteria were also frequently found (Zhang et al., 2012; Tang et al., 2013; Gomez-Saez et al., 2017). Despite nearly 30 published studies on shallow-water hydrothermal systems, many open questions remain about the chemosynthetic and photosynthetic microbes, including the metabolic pathways they use, how the pathways are coupled with each other, and what factors control their ecology.

Shallow (water depth < 30 m) submarine hydrothermal activity has been observed within 1 km east of Kueishantao Islet, off Taiwan. This hydrothermal system has unique geochemical characteristics and is driven by both sunlight and geothermal energy; thus, it is an ideal ecosystem to study coupled metabolic pathways and microbially driven biogeochemical cycles in extreme environments. Gas emitted from the Kueishantao hydrothermal vents are composed of CO_2_, nitrogen (N_2_), methane (CH_4_) and small amounts of hydrogen sulfide (H_2_S) (Chen et al., 2005; Chen et al., 2016). The hydrothermal fluids originate with deep magmatic matter and meteoric water from the Kueishantao Islet (Liu et al., 2010), and mix with seawater to form the final hydrothermal fluids. Fractures are widely developed around the andesite-hosted hydrothermal vent and therefore relatively oxygen-rich seawater seeps through these fractures in the seafloor. A previous study indicated that H_2_S in the Kueishantao hydrothermal system mainly originates from thermal reductive reactions of seawater and sulfate radicals, suggesting that seawater is the initial source of H_2_S (Zhang, 2013). Thus, steep geochemical gradients form when reduced hydrothermal fluids meet the oxidized seawater. Electron donors in the gradients include sulfur (S^0^), thiosulfate (S_2_O_3_^2-^), hydrogen (H_2_), organics, formate and fumarate, while nitrate, oxygen (O_2_), S^0^, and S_2_O_3_^2-^ are the major identified electron acceptors (Xie et al., 2011; Anantharaman et al., 2016; Tang et al., 2018). Thus, a series of redox reactions occur and drive the carbon, nitrogen, and sulfur cycles in this hydrothermal ecosystem, which consists of the vent fluids and the water surrounding the vent.

In this study, high-throughput 16S rRNA sequencing and metatranscriptome analyses were carried out to investigate the microbial community in the surface water immediately above a white hydrothermal vent and the bottom water next to the vent (Supplementary Figure S1). The potentially metabolically active bacterial compositions and metabolic pathways in the hydrothermal ecosystem were determined to improve our understanding of biogeochemical processes mediated by microorganisms and coupling of the carbon, sulfur, and nitrogen cycles in the water column of this unique ecosystem, driven by both sunlight and geothermal energy.

## Materials and Methods

### Study Sites and Sampling

A cluster of shallow hydrothermal vents is located within 1 km east of Kueishantao Islet (Supplementary Figure S1). A white vent was identified by scuba divers and its position was located using a Global Positioning System (24.83N, 121.96E). Two samples were collected in April 2014 from the surface water immediately above the vent (SW) and bottom water next to the vent (BW). All necessary permits were obtained for the described field studies, including the permits from the Coast Guard Administration of Taiwan and the Fisheries Management Office of the Yilan County.

On board, approximately 15 L of water per sample was pre-filtered through 3 μm pore-size polycarbonate membranes (EMD Millipore Corp., Darmstadt, Germany) and then collected in 0.22 μm Sterivex filter units (EMD Millipore Corp., Darmstadt, Germany) at a pressure of <0.03 MPa. The filtration was finished within 30 min to limit RNA degradation. As we could not assess whether the *in situ* expression profile had changed, a shorter filtering time (e.g., 10-15 min) may have been more appropriate. Each Sterivex was filled with RNA*later* RNA stabilization solution (Ambion, United States), flash frozen and stored in liquid nitrogen until RNA extraction.

### Biogeochemical Analysis

Salinity was obtained by the conversion of conductivity measurements from a Guildline salinometer (Autosal 8400B, Canada), and *in-situ* temperatures were determined by scuba divers using a thermocouple. pH values were measured with a pH meter (Radiometer PHM-85, Denmark) at 25°C and total alkalinity (TA) was measured with an alkalinity titrator. Nitrate, nitrite, and silicate were measured using a flow injection analyzer; the pink azo dye method was employed for nitrate and nitrite, and the silicomolybdenum blue method was used for silicate (Parsons, 1984; Pai et al., 1990). Dissolved inorganic carbon (DIC) was measured using a dissolved inorganic analyzer (AS-C3, Apollo SciTech, United States) with a precision of 0.1%.

### RNA Extraction, PCR Amplification, and Sequencing

Total RNA was extracted using TRIzol reagent (Invitrogen, United States) according to Simms et al. (1993) with minor modifications, and treated with DNase (Qiagen, Valencia, CA, United States). DNA contamination was checked by amplifying the bacterial 16S rRNA genes with the universal primers 27F and 1492R. The purified RNA without DNA contamination was reverse transcribed to synthesize complementary DNA (cDNA) using the SuperScript^TM^ III First Strand Synthesis System kit with random primers (Invitrogen, United States) following the manufacturer’s specifications. The V3-V4 hypervariable region of bacterial 16S rRNA genes was amplified with primers 343F and 798R (Wilson et al., 1990; Rochelle et al., 1995). The forward primer 343F (bold letters) contained transposase sequences (underlined letters): TCGTCGGCAGCGTCAGATGTGTATAAGAGACAG**TACGGRAGGCAGCAG**. The reverse primer 798R contained transposase sequences: GTCTCGTGGGCTCGGAGATGTGTATAAGAGACAG**AGGGTATCTAATCCT**. PCR reactions were conducted in triplicate. The 25 μL reactions contained 12.5 μL of 2 × KAPA HiFi Hotstart ReadyMix (Kapa Biosystems), 0.2 μM of each primer, and 2.5 μL of template cDNA (5 ng μL^-1^). The PCR thermal regime was initial denaturation at 95°C for 3 min, followed by 25 cycles of 30 s each of denaturation at 95°C, 30 s of annealing at 60°C, 30 s of extension at 72°C, and a final 10 min extension at 72°C. The products were purified with 0.8 volume of AMPure XP beads (Beckman Coulter, United States), and amplified with the P5 and P7 indexing primers. The products were further purified with 1 volume of AMPure XP beads, and then quantified with a Qubit dsDNA HS assay kit (Invitrogen, United States). Equimolar amounts of the PCR amplicons were pooled. Sequencing was conducted on an Illumina MiSeq sequencing system with MiSeq Reagent Kit v3 (Illumina, San Diego, CA, United States).

Raw data were filtered with MOTHUR v.1.32.1 (Schloss et al., 2009) for quality control. Paired-end DNA sequencing reads were chosen through the MOTHUR’s *make.contigs* routine and then assembled according to the overlap with no more than 1 base pair (bp) mismatch. Reads that either (1) contained more than one ambiguous nucleotide, (2) were shorter than 200 bp, or (3) had mononucleotide repeats greater than 8 bp were removed by *screen.seqs* routine. Sequences were clustered into operational taxonomic units with 97% similarity and the greengene database was used as a reference for taxonomic classification (Desantis et al., 2006). Based on the operational taxonomic unit assignment, diversity estimates (ACE, Chao 1, Shannon) were calculated using MOTHUR’s *summary.single* routine.

### mRNA Purification, Sequencing, Assembly, and Annotation

The Ribo-Zero Magnetic Gold kit (Epicenter, Madison, WI, United States) was used to remove rRNA from the purified RNA without DNA contamination and obtain mRNA. Fragmentation buffer (Ambion, United States) was added to digest the mRNA to produce short fragments. Using these short fragments as templates, the first-strand cDNA was synthesized with random hexamer primers. The second-strand cDNA was synthesized using the first-strand cDNA as a template, then further purified with the QiaQuick PCR Purification kit (Qiagen, Valencia, CA, United States), and dissolved with elution buffer for end reparation and adding poly (A). After that, the short fragments were connected with sequencing adapters. Finally, the second-strand cDNA was degraded with an enzyme (UNG), and the product purified with a MiniElute PCR Purification kit (Qiagen, Valencia, CA, United States) and sequenced with an Illumina HiSeq2000 (Illumina, San Diego, CA, United States).

After removing low quality reads with 10% N, 20% low quality bases (<Q20) contaminated by adapter, or rRNA (Li et al., 2009), *de novo* assembly of the obtained clean reads was performed using Trinity (Grabherr et al., 2011). These sequences were further assembled using the sequence clustering software TGICL (Pertea et al., 2003) to obtain longer and non-redundant unigenes. For gene family clustering, the merged unigenes were classified into one cluster when the similarity between them was >70%. The merged unigenes were then blasted against public databases, including the National Center for Biotechnology Information non-redundant protein sequence database and Kyoto Encyclopedia of Genes and Genomes (blastx, *E* < 10^-5^). The protein with the highest sequence similarity was retrieved and annotated to each unigene. For annotated unigenes, protein coding sequence information was retrieved from the blast results. For unannotated unigenes, ESTscan was used to perform protein coding sequence predictions. The predicted protein coding sequences were then blasted (blastp, *E* < 10^-5^) against databases, including eggNOG (Huerta-Cepas et al., 2016), the Carbohydrate Active Enzyme database (Cantarel et al., 2009), and the Antibiotic Resistance Genes database (Liu and Pop, 2009), to obtain further functional information. To assess the quality of assembly and facilitate the subsequent relative abundance analysis, reads of each sample were mapped back to the merged unigenes using Bowtie2 (Langmead and Salzberg, 2012). Only unique reads (aligned concordantly exactly one time) mapped to each unigene were considered for relative abundance analysis, and the number of hits identified in the read-mapping step was normalized as a percentage to the total mapped reads of the individual transcriptomic dataset.

## Results and Discussion

### Biogeochemical Conditions

Physicochemical parameters were measured at three depths in the water column and from the hydrothermal vent fluids (Table 1 and Supplementary Figure S2). The water column pH values ranged from 6.24 to 7.05 because they were influenced by the acidic hydrothermal vent fluids (pH 5.34). Temperatures varied from 23.6°C to 24.1°C, and were much lower than the vent fluids (48°C). Salinity (34.19–34.52) and DIC concentrations (2028-2040 μmol L^-1^) were almost constant within the water column, but lower and higher, respectively, in the vent fluids (salinity: 33.76; DIC: 2352 μmol L^-1^). Total alkalinity ranged from 1637 to 2038 μmol L^-1^, with the minimum in the BW. The nitrate and nitrite concentrations varied from 0.14 to 2.86 μmol L^-1^ and 0.03 to 0.32 μmol L^-1^, respectively. The silicate concentrations decreased with depth, ranging from 5.3 to 15 μmol L^-1^. The phosphate concentrations were below detection limit. Overall, the shallow-water hydrothermal ecosystem was characterized by acidic vent fluids that were well mixed with seawater. Sulfide (S^2-^) concentrations were not measured in this study. Our previous studies suggested that compared with deep-sea vents, sulfide was relatively lower in this shallow vent system, varying from 0.01 to 0.85 mg L^-1^ (Zhang et al., 2012). Tang et al. (2013) also reported a similar range of sulfide concentrations from 0.11 mg L^-1^ in the water in the vent to 0.01 mg L^-1^ in the surface water.

**Table 1:** Physicochemical parameters of the hydrothermal ecosystem in this study.

Sample	Depth (m)	pH	Temperature (°C)	Salinity	DIC (μmol L^-1^)	TA (μmol L^-1^)	NO_3_^-^ (μmol L^-1^)	NO_2_^-^ (μmol L^-1^)	SiO_3_^2-^ (μmol L^-1^)
Surface (SW)	0	6.24	24.1	34.19	2028	1865	2.61	0.32	15
Middle	7	7.05	–	34.52	2040	2038	0.14	0.17	6.8
Bottom (BW)	15	6.78	23.6	34.46	2040	1637	2.86	0.25	5.3
Vent fluids	19	5.34	48	33.76	2352	1947	0.77	0.03	–

*DIC, dissolved inorganic carbon; TA, total alkalinity*.

### Phylogenetic Identification of Active Bacteria Communities

**FIGURE 1:**
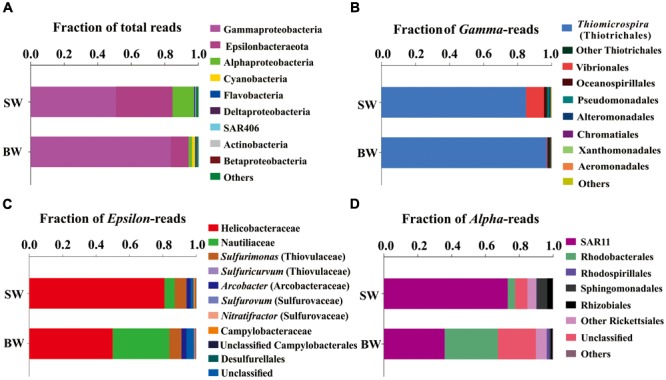
Phylogenetic taxon distribution among the bacterial RNA-based libraries. **(A)** Relative abundance of bacterial phyla or classes in total tags of each library. **(B)** Relative abundance of bacterial orders or genera in total gammaproteobacterial tags. **(C)** Relative abundance of bacterial orders, families, or genera in total epsilonbacteraeota tags. **(D)** Relative abundance of bacterial orders or clades in total alphaproteobacterial tags.

A total of 65,925 and 67,591 qualified reads were rendered from the 16S rRNA libraries of the SW and BW, respectively (Supplementary Table S1). Estimates of bacterial community diversity indicated that there were no significant differences between SW and BW (Supplementary Table S2). Ribotypes of tags were identified phylogenetically and grouped by phylum, order, family, or genus. Gammaproteobacteria and Epsilonbacteraeota were the overwhelmingly dominant groups in the water column of the hydrothermal ecosystem (Figure 1A). The sulfide-oxidizing *Thiomicrospira*, which belongs to Thiotrichales, was the most abundant group within Gammaproteobacteria (Figure 1B) as well as the total libraries with 81.69% of the total tags in the BW library and 43.5% in the SW library. The second most abundant group in the total libraries was the family Helicobacteraceae (mostly unclassified) in Epsilonbacteraeota (Figure 1C), with 27.34% of the total tags in the SW library and 5.24% in the BW library. At the genus level, sulfur-oxidizing bacteria *Sulfurimonas* (Thiovulaceae), *Arcobacter* (Arcobacteraceae), and *Sulfurovum* (Sulfurovaceae) were the most abundant within Epsilonbacteraeota (Figure 1C). Some sulfur-oxidizing bacterial species of the genus *Sulfurimonas* were also nitrate-reducing bacteria that accept electrons from the oxidation of reduced inorganic sulfur compounds (Waite et al., 2017). These autotrophic denitrifiers have been frequently identified from diverse ecosystems, such as deep-sea hydrothermal vents and the central Baltic Sea (Takai et al., 2005; Brettar et al., 2006). Sulfur oxidation coupled with dissimilatory nitrate reduction is usually an important source of energy for DIC fixation in hydrothermal vents (Shao et al., 2010). All but one of the *Sulfurimonas* species can also use H_2_ as an energy source (Han and Perner, 2014, 2015). Autotrophic sulfur-oxidizing Gammaproteobacteria and Epsilonbacteraeota were the dominant microorganisms in this shallow-water hydrothermal ecosystem, and may significantly contribute to primary production utilizing reduced sulfur compounds as electron donors (Brazelton and Baross, 2010; Campbell et al., 2013).

The family Nautiliaceae was the second most abundant group within Epsilonbacteraeota with 3.55% of the total tags in the BW library and 2.05% in the SW library (Figure 1C), and contains the genera *Lebetimonas* and *Nautilia* as well as a large number of unclassified taxa. Members of this family are moderate thermophiles growing optimally between 40 and 60°C (Nakagawa and Takai, 2014). Under autotrophic conditions, Nautiliaceae members have an ability to grow anaerobically via respiratory S^0^ reduction with H_2_, utilizing H_2_ as an electron donor and S^0^ and other reduced compounds as electron acceptors (Campbell et al., 2006; Meyer and Huber, 2014; Nakagawa and Takai, 2014).

The Alphaproteobacteria were mainly composed of the SAR11 clade, which was more abundant in SW than BW (Figure 1D). The abundance of SAR11 in shallow hydrothermal vents may be the result of vent fluids mixing with seawater, since the SAR11 clade is widely distributed in surface seawater (Morris et al., 2002). It has been reported that the SAR11 clade does not possess genes mediating assimilatory sulfate reduction and thus they would require exogenous reduced sulfur for their metabolism (Tripp et al., 2008). So, high reduced sulfur conditions may also explain SAR11 abundance in the Kueishantao hydrothermal ecosystem. Rhodobacteraceae, which belongs to Rhodobacterales, was the second most abundant Alphaproteobacteria in BW (Figure 1D). Rhodobacteraceae comprises mainly aerobic photoheterotrophs and chemoheterotrophs, as well as purple non-sulfur bacteria that perform photosynthesis in anaerobic conditions; they are deeply involved in sulfur and carbon biogeochemical cycling (Pujalte et al., 2014).

Cyanobacteria, assigned to *Synechococcus*, had a relative abundance of 0.12%-1.7% in the SW and BW 16S rRNA libraries (Figure 1A), which could be the result of vent fluids mixing with seawater. Cyanobacteria carry out oxygenic photosynthesis in the surface water and may switch to anoxygenic photosynthesis at vents where H_2_S is high (Cohen et al., 1975, 1986; Padan, 1979). Further studies are needed to verify this switch. These results indicate the co-occurrence of chemoautotrophs and photoautotrophs/heterotrophs in a shallow-water hydrothermal vent, which is distinctly different from deep-sea hydrothermal ecosystems (Tarasov et al., 2005).

Overall, the dominant active microorganisms in the shallow hydrothermal ecosystem were chemoautotrophic bacteria that mostly have the potential to perform sulfur oxidation and reduction, followed by phototrophs. Therefore, the ecosystem is driven by energy derived mainly from sulfur redox reactions and light. Hydrogen might also be an important energy source for this ecosystem, as sulfur-oxidizing *Sulfurimonas* species and sulfur-reducing Nautiliaceae species utilize H_2_ as an electron donor and they were found in the active assemblages (Muyzer et al., 1995; Brinkhoff et al., 1999). These results suggest multiple types of energy generation are performed in this system. Biogeochemical conditions to some extent determine the community composition and type of energy generation. Our previous study indicated that CH_4_ concentration was the statistically significant variable that explains the community structure in this shallow-water hydrothermal vent (Zhang et al., 2012). It is because distinctly different CH_4_ concentrations shaped different redox environments between the surface and bottom waters and consequently influenced the distribution of the community composition (Zhang et al., 2012). This is consistent with the results of this study where we found higher relative abundance of sulfur-reducing Nautiliaceae species in the BW library than the SW library. This result is also consistent with the finding of one order of magnitude higher sulfide concentration in the vent water than in the surface water (Tang et al., 2013).

### Major Metabolic Activities and Pathways

#### Carbon Fixation

**FIGURE 2:**
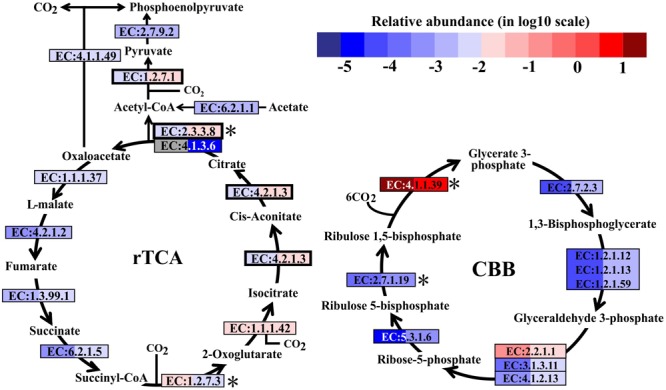
Carbon fixation pathways identified in the metatranscriptomes based on Kyoto Encyclopedia of Genes and Genomes pathway maps. Enzyme classification (EC) numbers for each step are included in boxes. The box color indicates relative abundance of reads in total mapped reads for each enzyme; red indicates higher relative abundance and blue lower relative abundance. The surface water (SW) is always shown on the left and bottom water (BW) on the right. Key enzymes are marked with an asterisk. A bold border indicates dual functional proteins. Gray represents absence. rTCA, reductive tricarboxylic acid cycle; CBB, Calvin-Benson-Bassham cycle.

In metatranscriptomes, 16,714 and 34,995 unigenes were obtained from SW and BW, respectively (Supplementary Table S3). Previous studies suggested that there are six pathways for CO_2_ fixation (Hügler and Sievert, 2011). In this study, all enzymes included in the Calvin-Benson-Bassham (CBB) and the reductive tricarboxylic acid (rTCA) cycles were detected (Figure 2). Overall, the relative transcript abundance of genes encoding enzymes in the rTCA cycle did not show a significant difference between the two samples (same order of magnitude). The relative transcript abundance of the key gene encoding ATP-citrate lyase (EC:2.3.3.8) in the rTCA cycle was nearly two times higher in BW. In contrast, another key gene (EC:1.2.7.3, 2-oxoglutarate synthase) had a slightly higher relative abundance in SW. However, the relative transcript abundance of genes encoding enzymes involved in the CBB cycle was one order of magnitude higher in BW, except for the genes encoding ribulose-1,5-bisphosphate carboxylase/oxygenase (RuBisCO) (EC:4.1.1.39) and transketolase (EC:2.2.1.1), which were one order of magnitude lower in BW (Figure 2). Moreover, the relative transcript abundance of genes encoding RuBisCO was distinctly higher (one to six orders of magnitude) than all other genes involved in the rTCA and CBB cycles. The rTCA cycle is universal in hydrothermal vent environments; it is performed by chemoautotrophic Epsilonbacteraeota (Hügler et al., 2005), and is the most economical pathway of carbon fixation in bacteria (Mangiapia and Scott, 2016). This pathway is particularly advantageous in an energy-limited environment (Oulas et al., 2016). The CBB cycle was the first carbon fixation pathway to be discovered about 60 years ago (Wilson and Calvin, 1955), and autotrophic Gammaproteobacteria mainly perform carbon fixation through this pathway (Hügler and Sievert, 2011).

#### Sulfur Cycle

**FIGURE 3:**
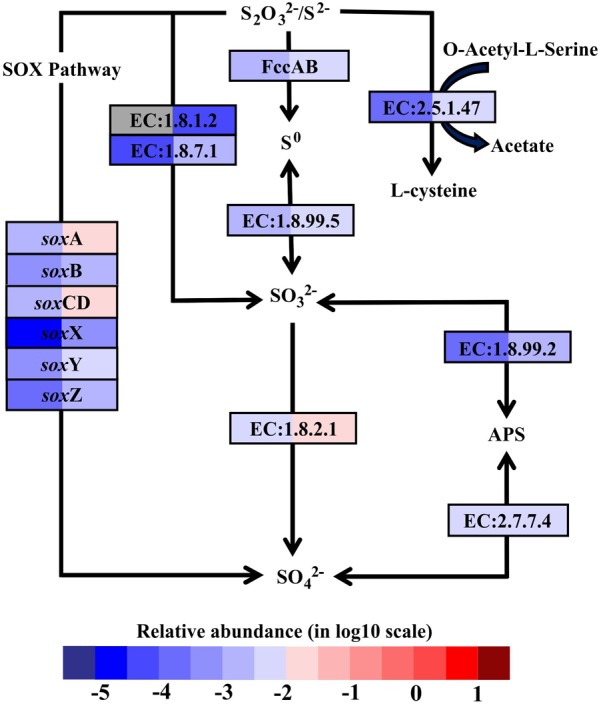
Sulfur metabolic pathways identified in the metatranscriptomes based on Kyoto Encyclopedia of Genes and Genomes pathway maps. Enzyme classification (EC) numbers for each step are included in boxes. The box color indicates relative abundance of reads in total mapped reads for each enzyme. The color scheme is the same as in Figure 2. Gray represents absence. SO_4_^2-^, sulfate; SO_3_^2-^, sulfite; S^0^, sulfur; S_2_O_3_^2-^, thiosulfate; S^2-^ sulfide; APS, adenylyl sulfate; FccAB, flavocytochrome c/sulfide dehydrogenase; SOX, thiosulfate oxidation multienzyme complex.

In hydrothermal ecosystems, sulfur redox reactions mediated by chemoautotrophic bacteria are the main processes for energy production and reliant upon hydrothermal vent fluids as an energy source (McCollom, 2000). In shallow-water hydrothermal vents, as metatranscriptome analyses revealed, oxidation of reduced sulfur compounds can play a key role in energy production. For example, gene encoding enzymes that catalyze sulfur compound oxidations from the most reduced sulfur species (–II) to the most oxidized inorganic form (+IV) were fully restored in the metatranscriptomes (Figure 3). The reverse sulfate reduction pathway includes the sulfide oxidation step involving flavocytochrome c sulfide dehydrogenase (FccAB), which initiates electron flow from sulfide to the transport chain. It also includes steps of sulfur oxidation to sulfite catalyzed by dissimilatory sulfite reductase (EC:1.8.99.5) and sulfite oxidation to sulfate catalyzed by sulfite dehydrogenase (EC:1.8.2.1). Additionally, sulfide/disulfide could be oxidized to sulfite catalyzed by assimilatory NADPH-sulfite reductase (EC:1.8.1.2) and ferredoxin-sulfite reductase (EC:1.8.7.1). The Sox pathway was another important sulfur oxidation pathway found in the Kueishantao white hydrothermal vent, as *sox*ABCDXYZ genes encoding the thiosulfate oxidation multienzyme complex were retrieved from the metatranscriptomes (Figure 3). In addition, the sulfate adenylyltransferase (EC:2.7.7.4) and adenylylsulfate reductase (EC:1.8.99.2) were also identified in both BW and SW; these are involved in the energy-yielding dissimilatory sulfate reduction pathway (Figure 3). These results are consistent with the active bacterial community compositions, in which a large fraction of sulfur-oxidizing bacteria and a small number of sulfate-reducing bacteria were retrieved from the shallow hydrothermal vent. Overall, the relative transcript abundances of genes encoding the enzymes involved in these sulfur redox reactions were one to two orders of magnitude higher in BW than SW. The only exceptions were the *sox*B gene and the gene encoding sulfate adenylyltransferase, which were comparable between the two samples.

#### Nitrogen Cycle

**FIGURE 4:**
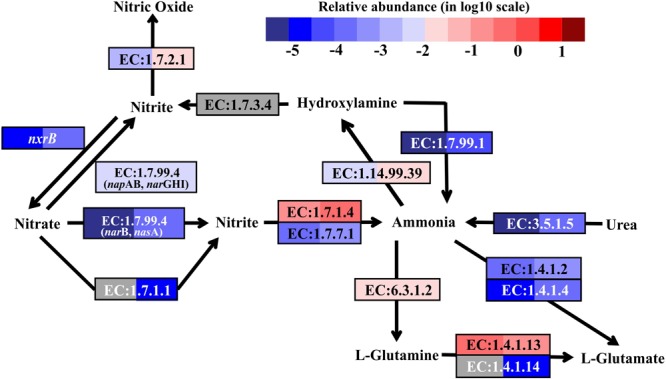
Nitrogen metabolic pathways identified in the metatranscriptomes based on Kyoto Encyclopedia of Genes and Genomes pathway maps. Enzyme classification (EC) numbers for each step are included in boxes. The box color indicates relative abundance of reads in total mapped reads for each enzyme. The color scheme is the same as in Figure 2. Gray represents absence.

Denitrification, coupled to sulfur oxidation, usually plays an important role in most hydrothermal vent environments (Bourbonnais et al., 2012, 2014; Voss et al., 2013; Dang and Chen, 2017). Genes encoding dissimilatory nitrate reductase (EC:1.7.99.4, *nap*AB and *nar*GHI) and nitrite reductase (EC:1.7.2.1, nitric oxide-forming), involved in denitrification, were found in the metatranscriptomes from both SW and BW (Supplementary Table S4). The transcripts of genes encoding assimilatory nitrate reductase (EC:1.7.99.4, *nar*B and *nas*A, and EC:1.7.1.1), NAD(P)H-nitrite reductase (EC:1.7.1.4), and ferredoxin-nitrite reductase (EC:1.7.7.1) were also retrieved from the metatranscriptomes (Figure 4). Nitrate and nitrite could be important electron acceptors when dissimilatory nitrate reduction (to ammonium, DNRA, or denitrification) is coupled to sulfur oxidation reactions (Nakagawa and Takai, 2008; Dang and Chen, 2017). For example, the sulfur and thiosulfate-oxidizing bacteria *Sulfurovum* sp., which were detected in the 16S rRNA libraries, have been reported to use nitrate or oxygen as electron acceptors (Inagaki et al., 2004; Giovannelli et al., 2016; Dang and Chen, 2017). In the present study, the transcripts of genes encoding nitrite reductase (ammonia-forming) related to DNRA (e.g., NirBD and NrfAH, according to the Kyoto Encyclopedia of Genes and Genomes) were not retrieved. Previous studies indicated that DNRA (nitrite ammonification) was mainly important in relatively reducing environments, as found in nutrient-rich coastal sediments (Rysgaard et al., 1996; Christensen et al., 2000; An and Gardner, 2002), while denitrification was more important in low to moderate organic-loading sediments (Fossing et al., 1995; Zopfi et al., 2001). For instance, a metagenomics analysis of sulfur-oxidizing Gammaproteobacteria from a coastal ecosystem in the eastern South Pacific suggested a coupling of sulfur oxidation and DNRA in oxygen-deficient waters (Murillo et al., 2014).

As nitrification marker genes, the transcripts of ammonia monooxygenase (*amo*ABC) genes (EC:1.14.99.39), responsible for ammonia oxidation to hydroxylamine, and nitrite oxidoreductase beta subunit (*nxr*B), responsible for nitrite oxidation to nitrate, were retrieved in the metatranscriptomes of SW and BW (Figure 4). Thus, there may be a coupling of nitrification and denitrification processes, resulting in nitrogen removal via the nitrite pathway.

In addition, the reduction of hydroxylamine and the hydrolysis of urea could produce ammonia in this shallow-water hydrothermal vent, as the genes encoding hydroxylamine oxidase (EC:1.7.99.1) and urease (EC:3.5.1.5) were identified in the metatranscriptomes (Figure 4). Ammonia can be stored in glutamine catalyzed by glutamine synthetase (EC:6.3.1.2) or glutamate catalyzed by glutamate dehydrogenase (EC:1.4.1.2 and EC:1.4.1.4). The former could be the main pathway, since the relative transcript abundance of the genes encoding glutamine synthetase was one to three orders of magnitude higher in SW and BW than glutamate dehydrogenase. Overall, the relative transcript abundances of genes encoding enzymes involved in the nitrogen cycle were up to one order of magnitude higher in BW. Detailed information on major carbon, sulfur, and nitrogen metabolic pathways are shown in (Supplementary Table S4).

### Main Players in Major Metabolic Pathways

**FIGURE 5:**
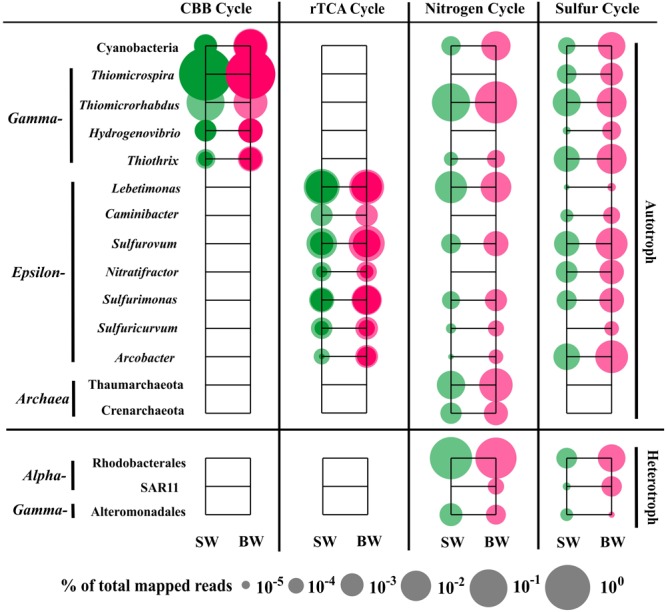
Distribution of relative transcript abundance of genes encoding enzymes included in carbon fixation, and nitrogen and sulfur metabolism among phylogenetic taxa. For the carbon fixation pathway, dark circles indicate reads of genes encoding key enzymes (marked with an asterisk in Figure 2); light circles indicate reads of genes encoding enzymes involved in each pathway. SW, surface water immediately above the vent; BW, bottom water next to the vent.

The sequences classified as Gammaproteobacteria, mainly *Thiomicrospira*, were the most abundant in the metatranscriptome dataset, which was consistent with the 16S rRNA libraries analysis that showed *Thiomicrospira* as having the highest abundance active population (Supplementary Table S5). Gene annotation and functional analysis indicated that the gammaproteobacterial genera *Thiomicrospira, Thiomicrorhabdus, Hydrogenovibrio*, and *Thiothrix*, Epsilonbacteraeota genera *Lebetimonas* and *Caminibacter* (Nautiliaceae), *Sulfurovum* and *Nitratifractor* (Sulfurovaceae), *Sulfurimonas* and *Sulfuricurvum* (Thiovulaceae), and *Arcobacter* (Arcobacteraceae), as well as Cyanobacteria (mainly *Synechococcus* and *Prochlorococcus*) and Archaea (Thaumarchaeota and Crenarchaeota) were the main autotrophic players in carbon fixation, and nitrogen and sulfur metabolism (Figure 5).

The most abundant transcript sequences belonged to *Thiomicrospira* (mainly *T. crunogena* XCL-2), most of which was involved in the CBB cycle (mainly RuBisCO sequences) (Figure 5). *Thiomicrospira crunogena* was originally isolated from the East Pacific Rise (Jannasch et al., 1985), and was subsequently detected in deep-sea hydrothermal vents (Wirsen et al., 1998) as well as shallow-water hydrothermal vents (Muyzer et al., 1995; Brinkhoff et al., 1999). It has been known as the representative ubiquitous chemolithoautotrophic sulfur-oxidizing bacteria (Scott et al., 2006) and has a remarkably high growth rate (Jannasch et al., 1985). In addition, the transcript sequences belonging to *Thiothrix* (mostly *T. nivea* DSM 5205), encoding RuBisCO, transketolase, and fructose-1,6-bisphosphatase in the CBB cycle, as well as FccAB and *sox*X, were retrieved from our metatranscriptome data. Thus, *T. nivea* could be chemolithoautotrophic via the CBB cycle or mixotrophic, since it has been reported to grow under heterotrophic conditions (Lapidus et al., 2011). A number of sequences classified as *Thiomicrorhabdus* within Gammaproteobacteria encoded assimilatory NAD(P)H-nitrite reductase and glutamine synthase, transketolase, and fructose-1,6-bisphosphate aldolase in the CBB cycle, as well as sulfate adenylyltransferase, FccAB, and *sox*ABY.

The transcript sequences within Epsilonbacteraeota were involved in the rTCA pathway, dissimilatory/assimilatory nitrate reduction, and sulfur oxidizing/reducing processes (Figure 5). Nautiliaceae is a typical sulfur-reducing bacterial family living in hydrothermal vents, which can get energy from the oxidation of H_2_ or formate coupled with reduction of S^0^, to produce H_2_S (Hanson et al., 2013; Nakagawa and Takai, 2014). In our metatranscriptomes, they were abundant and involved in dissimilatory nitrate reduction, but very few were involved in sulfate reduction. For example, *Lebetimonas* had the most abundant transcript sequences in the total sequences involved in dissimilatory nitrate reduction (89.1% in SW and 54.1% in BW). All sequences belonging to *Caminibacter* were classified to *C. mediatlanticus*. Although they have been identified as sulfur or nitrate-reducing bacteria (Voordeckers et al., 2005), the sequences retrieved from our metatranscriptomes mainly related to the oxidation of sulfide to sulfur and the rTCA pathways. *Sulfurovum* (Sulfurovaceae) are typical (not strictly anaerobic) sulfur-oxidizing bacteria that can utilize nitrate or oxygen as electron acceptors (Inagaki et al., 2004). *Nitratifractor*, previously belonging to Nautiliaceae, were reclassified to Sulfurovaceae (Waite et al., 2017). In our metatranscriptomes, all sequences belonging to *Nitratifractor* were classified to *N. salsuginis* and mainly related to sulfur oxidation and rTCA pathways. *Sulfurimonas denitrificans* is a nitrate-reducing, sulfur-oxidizing species, but other *Sulfurimonas* species sequences retrieved from our metatranscriptomes were involved in assimilatory nitrate reduction, ammonia incorporation to glutamine, and glutamate pathways. The sequences assigned to *Sulfuricurvum* are associated with dissimilatory nitrate reduction, glutamine and cysteine synthesis, dissimilatory sulfate reduction, and rTCA pathways, although *Sulfuricurvum* species were reported to be sulfur-oxidizing bacteria (Kodama and Watanabe, 2004). *Arcobacter* had the most abundant transcript sequences in total sequences involved in sulfur metabolism, encoding sulfite dehydrogenase, FccAB, *sox*ABCDXYZ, and cysteine synthase. Moreover, in our metatranscriptomes, hydrogenase sequences were identified in sulfur-reducing *Lebetimonas* and sulfur-oxidizing *Sulfurimonas* and *Sulfuricurvum* (Supplementary Table S6), suggesting that they might obtain energy through the oxidation of hydrogen.

Notably, Cyanobacteria (mainly *Synechococcus* and *Prochlorococcus*) participated in carbon (CBB cycle), nitrogen (ammonia assimilation), and sulfur (sulfide oxidation and sulfate assimilation) metabolism, although they were less abundant in the active population of the two 16S rRNA libraries (Figure 5 and Supplementary Table S5). Most Cyanobacteria are highly sensitive to sulfide toxicity (Oren et al., 1979), but some species are sulfide resistant (Cohen et al., 1986) or perform anoxygenic photosynthesis using sulfide rather than water as the terminal reductant (Frier et al., 1999; Kulp et al., 2008; Stal, 2012). A recent study indicated that under darkness and anoxygenic conditions, hydrogen in H_2_S accelerated the recovery of photosynthesis, and even enhanced photosynthetic rates at a given H_2_S concentration at low irradiance (Klatt et al., 2015). The geochemical properties of shallow-water hydrothermal environments possibly have retained many of the characteristics of the Earth’s early ocean (Baross and Hoffman, 1985) and thus Cyanobacteria in the habitat studied here may also preserve some of these characteristics. In addition, archaeal sequences were also retrieved, and belonged to Thaumarchaeota and Crenarchaeota, which were only involved in the nitrogen cycle, including *amo*ABC and nitrite reductase (*nir*K). Sequences associated with the archaeal carbon fixation pathway, 3-hydroxypropionate/4-hydroxybutyrate (HP/HB) cycle, were not found (Figure 5).

Most of the reads within Alphaproteobacteria were associated with organic carbon metabolism and belonged to Rhodospirillales. However, the most abundant transcript sequences within Alphaproteobacteria involved in nitrogen and sulfur metabolism (sulfur oxidation, cysteine synthesis, and glutamine/glutamate synthesis) belonged to the order Rhodobacterales (Figure 5). Moreover, the transcript sequences encoding bacteriochlorophyll synthase were retrieved and assigned to *Ahrensia*, which is a typical aerobic photoheterotrophic genus in Rhodobacteraceae. The reads belonging to the SAR11 clade only accounted for ∼2% in SW and ∼7% in BW in our metatranscriptomes, despite the high proportions within Alphaproteobacteria in the two 16S rRNA libraries (∼73% in SW and ∼36% in BW); these transcript sequences are associated with glutamine and cysteine synthesis and dissimilatory sulfate reduction pathways. The most abundant transcript sequences within heterotrophic Gammaproteobacteria involved in nitrogen and sulfur metabolism belonged to Alteromonadales (Figure 5).

### Stress Tolerance

Most genes related to stress tolerance showed higher relative transcript abundance in BW, including genes encoding multiple molecular chaperones, anaerobic regulator proteins, and various antioxidant genes (Supplementary Figure S3). In addition, the relative transcript abundances of genes encoding DNA mismatch repair proteins were high in both SW and BW (Supplementary Figure S3). The DNA mismatch repair system plays a vital role in an organism’s response to DNA damage and maintains genomic stability. These results suggest that the microbial communities have evolved extensive DNA repair systems, such as heat shock stress response systems and regulators of anaerobiosis or antioxidant systems, to cope with the extreme conditions present at a hydrothermal vent (Kiley and Beinert, 1998; Xie et al., 2011).

## Conclusion

High-throughput 16S rRNA sequences and metatranscriptome analyses revealed that Gammaproteobacteria and Epsilonbacteraeota were the most active bacterial populations involved in the major carbon, sulfur, and nitrogen metabolic pathways in a shallow-water hydrothermal ecosystem. The major sulfur oxidizers were *Thiomicrospira, Thiomicrorhabdus*, and *Thiothrix* from Gammaproteobacteria, and *Arcobacter* and *Sulfurovum* from Epsilonbacteraeota, which showed high transcript abundances in the genes involved in the SOX/reverse sulfate reduction pathway. The sulfur-reducing Nautiliaceae contributed very few transcripts to sulfate reduction, but showed a high level of transcription for genes involved in denitrification processes. In addition, *Thiomicrorhabdus* exhibited a range of genes related to assimilatory nitrate reduction.

**FIGURE 6:**
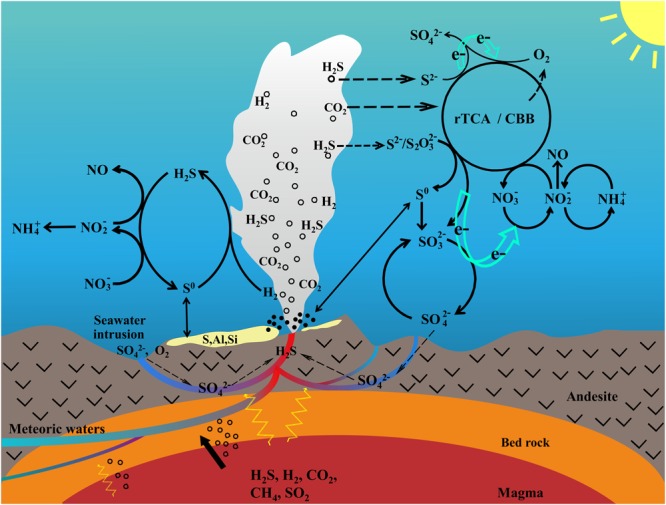
Schematic diagram illustrating the coupling of the carbon, sulfur, and nitrogen cycles mediated by microorganisms in the shallow-water hydrothermal ecosystem. SO_4_^2-^, sulfate; SO_3_^2-^, sulfite; S/S^0^, sulfur; S_2_O_3_^2-^, thiosulfate; S^2-^, sulfide; H_2_S, hydrogen sulfide; SO_2_, sulfur dioxide; NO_3_^-^, nitrate; NO_2_^-^, nitrite; NH_4_^+^, ammonium; NO, nitric oxide; H_2_, hydrogen; CH_4_, methane; CO_2_, carbon dioxide; O_2_, oxygen.

We illustrated these major metabolic pathways and the possible coupling between microbially driven biogeochemical cycles in this ecosystem (Figure 6). Hydrogen sulfide contained in hydrothermal fluids from the Kueishantao vents is produced from thermal reduction of seawater sulfate radicals when seawater seeps through fractures in the seafloor (Figure 6). This suggested that seawater is the initial source of H_2_S and geothermal heat is the primary energy source. Consequently, chemolithoautotrophic microbes (mainly members within Gammaproteobacteria and Epsilonbacteraeota) derive energy from the oxidation of reduced sulfur compounds and fix DIC by the CBB and rTCA cycles. Sox-dependent and reverse sulfate reduction are the main pathways of energy generation, and are probably coupled to denitrification by the provision of electrons to nitrate and nitrite (Figure 6). Oxygen is also a possible electron acceptor for sulfur oxidation. In addition, hydrogen oxidation supplies metabolic energy for some sulfur-oxidizing (e.g., *Sulfurimonas* sp.) and sulfur-reducing (e.g., *Lebetimonas* sp.) bacteria, coupled to the reduction of nitrate and sulfur, respectively. Ammonia and nitrite oxidation are other types of energy generation carried out by Thaumarchaeota/Crenarchaeota and *Nitrospina*, respectively, in this hydrothermal system, and coupled to denitrification by providing nitrate and nitrite substrate (Figure 6). Furthermore, driven by light energy, Cyanobacteria and aerobic photoheterotrophs also actively participate in major metabolic pathways. We speculate that Cyanobacteria perform oxygenic photosynthesis, fixing CO_2_ through the CBB cycle and producing O_2_ in the surface water, and then switch to anoxygenic photosynthesis fixing CO_2_ and producing S^0^ or sulfite in the bottom water next to the vent (Figure 6). Our results indicate the co-occurrence of chemoautotrophs and photoautotrophs/heterotrophs in a shallow-water hydrothermal vent, which is distinctly different from deep-sea hydrothermal ecosystems. Overall, the oxidation of reduced sulfur compounds, using oxygen or nitrate as electron acceptors, provide significant energy for carbon fixation in this shallow-water hydrothermal vent ecosystem, which uses sunlight and geothermal as primary energy sources. This study helps us to better understand biogeochemical processes mediated by microorganisms and the possible coupling of the carbon, sulfur, and nitrogen cycles in this unique ecosystem.

## Data Availability

The 16S rRNA sequence and metatranscriptomic datasets were deposited in the Short Reads Archive (National Center for Biotechnology Information) under accession numbers SRP145248 and SRP145422.

## Author Contributions

YZ conceived and designed the research. YL, KT, LZ, ZZ, XX, C-TC, and DW conducted the experiments. YL, KT, YZ, LZ, ZZ, XX, C-TC, and NJ analyzed the data. YL, KT, LZ, and YZ wrote the paper. NJ, C-TC, and DW contributed to the interpretation of results and critical revision.

## Conflict of Interest Statement

The authors declare that the research was conducted in the absence of any commercial or financial relationships that could be construed as a potential conflict of interest.
