# Supplementary material for: Coupled Carbon, Sulfur, and Nitrogen Cycles Mediated by Microorganisms in the Water Column of a Shallow-Water Hydrothermal Ecosystem
Source: Front Microbiol. 2018 Nov 13;9:2718. doi: 10.3389/fmicb.2018.02718 (PMC6282030; doi:10.3389/fmicb.2018.02718)
Supplement: Supplementary file 1 [file Data_Sheet_1.DOCX]

**Supporting Information**

**Figure S1.** Sampling locations and depths, and photographs of the white hydrothermal vent. SW, the surface water immediately above the vent; BW, the bottom water beside the vent.

**Figure S2.** Physicochemical parameters of the hydrothermal ecosystem in this study. DIC, dissolved inorganic carbon; TA, total alkalinity.

**Figure S3.** Relative transcript abundance of genes encoding enzymes associated with a stress response.

**Table S1.** Qualified reads from the 16S rRNA libraries of the SW and BW samples.

**Table S2.** Diversity estimates of the two samples (cut off at 0.01‒0.05).

**Table S3.** Assembly result statistics of the metatranscriptome reads.

**Table S4.** Detected sequences for genes related to carbon fixation, and sulfur and nitrogen metabolism in metatranscriptomes. (See *Supplementary_Data Sheet 2.xlsx*)

**Table S5.** Taxonomic breakdown of the main groups involved in carbon fixation, and sulfur and nitrogen metabolism in metatranscriptomes. (See *Supplementary_Data Sheet 3.xlsx*)

**Table S6.** Detected sequences for genes related to hydrogen oxidation in metatranscriptomes. (See *Supplementary_Data Sheet 4.xlsx*)

**Figure S1**


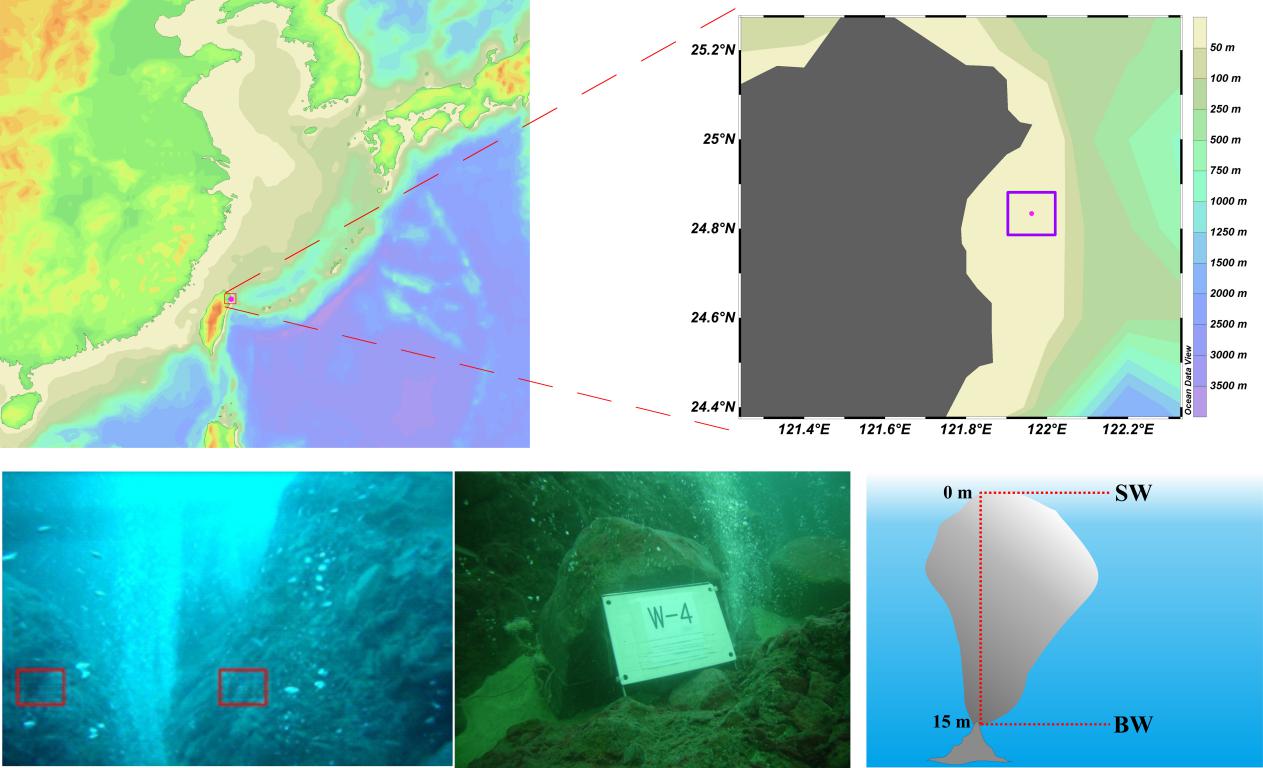


**Figure S2**


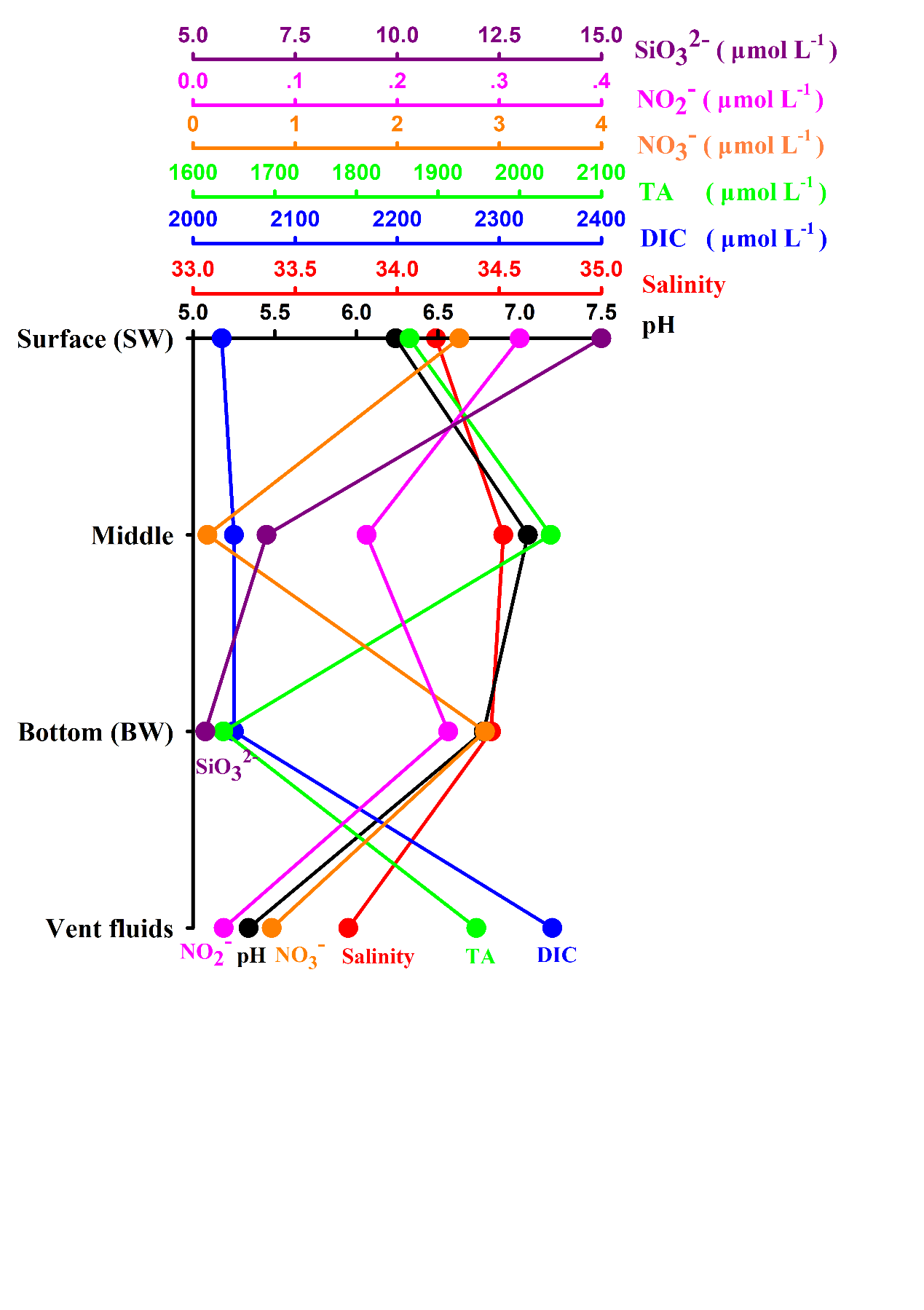


**Figure S3**

**
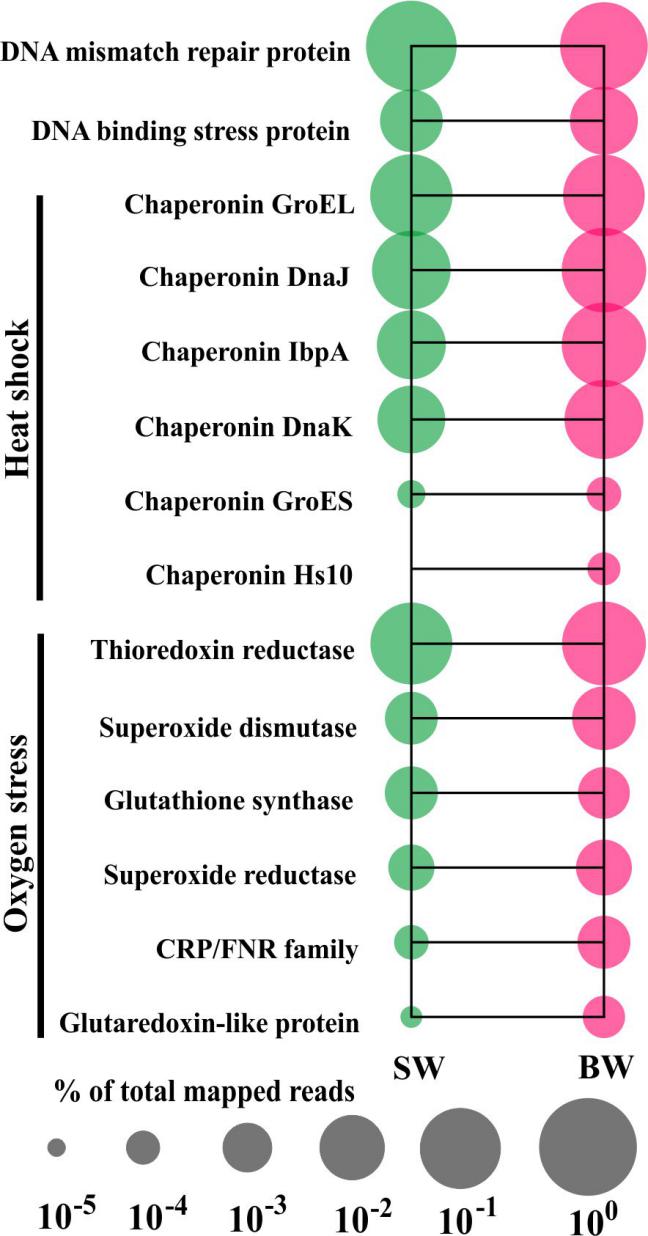
**

| **Table S1.** Qualified reads from the 16S rRNA libraries of the SW and BW samples | | | | |
| --- | --- | --- | --- | --- |
| **Sample** | **Number** | **Min length (bp)** | **Max length (bp)** | **Average length (bp)** |
| SW | 65,925 | 235 | 443 | 413 |
| BW | 67,591 | 236 | 462 | 422 |

| **Table S2.** Diversity estimates of the two samples (cut off at 0.01‒0.05) | | | | | | | |
| --- | --- | --- | --- | --- | --- | --- | --- |
| **Sample** | **Cutoffs** | **OTUs** | **ACE** | **Chao** | **Shannon** | **Simpson** | **Coverage** |
| SW | 0.01 | 6697 | 45665.605 | 23675.803 | 3.949903 | 0.153078 | 0.923595 |
| SW | 0.02 | 3797 | 16811.932 | 11703.858 | 3.752212 | 0.153546 | 0.963549 |
| SW | 0.03 | 2545 | 12557.274 | 8252.5826 | 3.571393 | 0.154574 | 0.976064 |
| SW | 0.04 | 1908 | 9134.9537 | 5787.5028 | 3.361598 | 0.160028 | 0.982116 |
| SW | 0.05 | 1605 | 6537.426 | 4112.6983 | 3.249669 | 0.162882 | 0.98562 |
| BW | 0.01 | 7970 | 53188.71 | 27426.142 | 2.932143 | 0.456799 | 0.910609 |
| BW | 0.02 | 4456 | 19716.011 | 12322.383 | 2.717695 | 0.456971 | 0.957716 |
| BW | 0.03 | 2884 | 12156.296 | 7168.5698 | 2.521493 | 0.458424 | 0.974079 |
| BW | 0.04 | 2020 | 7365.9443 | 4768.5 | 2.257511 | 0.474436 | 0.982305 |
| BW | 0.05 | 1611 | 5058.9047 | 3526.7243 | 2.131708 | 0.482625 | 0.986596 |

| **Table S3.** Assembly result statistics of the metatranscriptome reads | | | | | |
| --- | --- | --- | --- | --- | --- |
|  | **Sample** | **Total number** | **Total length (nt)** | **Mean length (nt)** | **N50** |
| Contig | SW | 166,606 | 28,374,051 | 170 | 155 |
|  | BW | 437,843 | 66,382,659 | 152 | 142 |
| Unigene | SW | 16,714 | 10,168,430 | 608 | 957 |
|  | BW | 34,995 | 16,826,910 | 481 | 533 |
